# Improving Criminal Responsibility Determinations Using Structured Professional Judgment

**DOI:** 10.3389/fpsyg.2021.700991

**Published:** 2021-07-13

**Authors:** Marvin W. Acklin, Joseph P. Velasquez

**Affiliations:** ^1^Department of Psychiatry, John A. Burns School of Medicine, University of Hawaii at Mānoa, Honolulu, HI, United States; ^2^Department of Psychology, Chaminade University of Honolulu, Honolulu, HI, United States

**Keywords:** criminal responsibility, forensic assessment, structured professional judgment, actuarial prediction, methamphetamine psychosis

## Abstract

Forensic psychologists commonly utilize unstructured clinical judgment in aggregating clinical and forensic information in forming opinions. Unstructured clinical judgment is prone to evaluator bias and suboptimal levels of inter-rater reliability. This article proposes Structured Professional Judgment (SPJ) methods as a potential remedy. Following a review of canonical forensic assessment models, the prevalence of bias in forensic judgments, and inter-rater agreement in criminal responsibility (CR) determinations, this article presents a SPJ model for CR evaluations translated from violence risk assessment methodology. A systematic user-friendly methodology is described, applying procedural checklists, application of a mental state at time of the offense (MSO) model using structured data collection methods, aggregation of empirical evidence guidelines, and *post-hoc* hypothesis testing using the Analysis of Competing Hypotheses (ACH). A case study describes application of the procedural and CR decision model in a complex homicide case. The model demonstrates the power and efficacy of the application of SPJ to forensic decision-making and is relevant to other types of forensic assessment (e.g., competency to stand trial, post-acquittal release decision-making).

## Criminal Responsibility Evaluations

Criminal responsibility (CR) evaluations are complex forensic mental health evaluations requiring collecting, aggregating, and interpreting data from multiple sources (e.g., Rogers and Shuman, 2000; Melton et al., 2007; Acklin, 2008). Based on relevant legal standards, the examiner must engage in a multi-step sequential process: frame investigative hypotheses, collect data, establish a threshold clinical diagnosis, make a determination of the mental state at the time of the offense (MSO), aggregate data into a decision model, and render an opinion linking clinical data and legal standard (Grisso, 2003)^1^. These decisions are typically be made using clinical (holistic or informal) judgment methods (Dawes et al., 1989).

Over the last 60 years, the clinical vs. statistical debate has been ongoing. Meehl (1954) originally defined *clinical judgment* as an informal, subjective, non-quantitative mode of aggregating observations to make predictions. Informal or holistic aggregation of data is prone to judgmental biases and heuristics identified, for example, by Tversky and Kahneman (1974). A significant literature has described the short comings of clinical judgment in clinical and forensic decision-making (Faust and Ahern, 2012). Studies have demonstrated the superiority of decision-making utilizing Structured Professional Judgment (SPJ; Hart et al., 2016) and actuarial, mechanical, or algorithmic judgment methods over unstructured clinical judgment (Grove and Meehl, 1996; Grove et al., 2000).

Surveys indicate that most clinicians rely on unstructured clinical judgment in both clinical and forensic work. The predilection for unstructured clinical judgment in forensic decision-making prone to biases, heuristics, and error has provoked intense discussion in forensic psychology (Neal and Grisso, 2014a,b). The next section will examine canonical models for forensic psychological evaluations in light of the clinical-actuarial judgement controversy.

## Canonical Assessment Models in Forensic Psychology

Many commentators and critics of clinical and forensic judgment advocate for a transparent and structured approach to data gathering, aggregation, and judgment. Grisso (2003) specified components that legal competency evaluations have in common, including: (a) functional, (b) causal, (c) interactive, (d) judgmental, and (e) dispositional components. The interactive component requires a judgment about the individual's level of capacity to meet the demands of the specific situation; specifically identifying the incongruence between a person's functional ability and the degree of performance demanded by the specific context. The judgmental and dispositional components require a judgment that the person-context incongruence is of sufficient magnitude to warrant a finding of legal significance.

Heilbrun et al. (2003) formulated a set of 29 principles that serve as a conceptual and procedural framework for forensic mental health assessment (FMHA) procedures. In the effort to improve the quality of forensic reports they recommend standardization of procedures and report elements. Their proposed model includes (a) clearly stated referral question; (b) coherent report organization; (c) elimination of jargon; (d) inclusion of data relevant to forensic opinion; (e) separation of observations from inferences; (f) consideration of multiple data sources, if possible; (g) appropriate use of psychological testing; (h) consideration of alternate hypotheses; (i) data-supported opinions; and (j) and clear linkage between data and opinions. Empirical study of Heilbrun's structured principles found modest improvements in report quality and relevance (Lander and Heilbrun, 2009) but rather poor adherence to the assessment principles. Significantly, Heilbrun's model does not specify principles or procedures for data aggregation or hypothesis testing. The model as described relies on informal aggregation and unstructured clinical judgment in the linkage between data and opinions.

It is unlikely that procedural standardization alone will be sufficient to correct biases associated with unstructured clinical judgment. Forensic evaluators have been shown to utilize the same biases and heuristics common in non-professionals (Lilienfeld and Lynn, 2015). It should not be surprising that levels of agreement are poor given the complexity of forensic evaluations and the widespread reliance on unstructured clinical judgment in forensic decision making (Monahan, 2008; Sutherland et al., 2012; Hart et al., 2016).

Selection and confirmation biases may enter into the evaluation process at multiple points during the course of the evaluation. Methods for collection, aggregation, and interpretation of information are not typically described. The “gap” between data and forensic opinion is a critical juncture in decision making process (Hart et al., 2016). Even highly skilled mathematical psychologists are unsophisticated in computational decision making and utilize heuristics in addressing simple problems (Kahneman and Frederick, 2002). Review of the various authorities do not provide guidance on methods for integrating data into inferences and opinions.

Evaluators are typically advised to apply informal additive or summative models of data aggregation in opinion formation. Principle 22 of Heilbrun's model may serve as an example. “Use scientific reasoning in assessing causal connection between clinical condition and functional abilities” (Heilbrun et al., 2003, p. 335). Evaluators are advised to “describe explanations for clinical condition and functional abilities that have the most supporting evidence and least disconfirming evidence” (p. 335). The AAPL Practice Guidelines for the conduct of insanity evaluations (2014) are even less specific, advising the forensic evaluator to “consider to what degree the mental condition and its relationship to the alleged crime meets the legal standard for criminal responsibility.” The reliance on *ad-hoc* clinical judgment is prone to intuitive heuristics and various biases has been severely criticized by advocates of actuarial or algorithmic decision making (Dawes et al., 1989; Hilton et al., 2006; Falzer, 2013).

## Bias in Forensic Mental Health Evaluations

Following the pioneering work of Tversky and Kahneman (1974), Neal and Grisso (2014a,b) describe a detailed variety of cognitive heuristics in forensic psychological assessment, including the representativeness (conjunction fallacy, base rate neglect) and availability heuristics (confirmation bias and what you see is all there is), and anchoring bias (biased thinking tied to initial premises). They advocate general remedies without procedural specification: hypothesis testing procedures, structured methods for forensic assessment, and application of actuarial measures over unstructured clinical judgment as methods to improve reliability. When surveyed about bias in forensic mental health evaluations, experts assert that “will power” and “introspection” are potential correctives to biased thinking. Evaluators acknowledge bias in their peer's judgments more than their own (blind spot bias). Evaluators perceive themselves as less subject to bias than their colleagues (Neal and Brodsky, 2016; Zapf et al., 2018).

Murrie and colleagues identified “adversarial allegiance” as an additional source of bias, namely, the tendency to skew scores and interpretations on forensic assessment instruments based on allegiance to the retaining party (Murrie et al., 2008, 2009, 2013). These reports sent shock waves through the FMHA community. It is not at all clear how or whether the publication of these findings has had any appreciable effect on forensic practice since practitioners are resistant to modifications of practice and are disinclined to utilize structured assessment methodologies (Vrieze and Grove, 2009; Lilienfeld et al., 2013).

## Interrater Reliability in Clinical and Forensic Decision Making

Interrater reliability is a useful performance indicator for the efficiency, accuracy, and reproducibility of forensic judgments. “Analyses of agreement between clinicians can be of value in examining accuracy” (Faust and Ahern, 2012, p. 151). The reliability of a measure is indicative of the reproducibility of the judgment, the degree of true variance, confidence that can be placed on judgments, and the degree of error that will be introduced into the decision making task (Kraemer et al., 2002). High levels of reliability, however, are a necessary (but not sufficient) indicator of accuracy. Poor or even marginal reliability raises concerns about bias, inaccuracy, and error. The following section examines interrater reliability for two necessary components of the CR evaluation—clinical assessment for the MSO, including psychiatric diagnosis and forensic opinion.

### Clinical Diagnostic Decisions

The reliability of psychiatric diagnosis has been a constant concern since the emergence of the DSMs. Clinical assessment focuses on psychiatric symptoms and diagnoses as a threshold condition for MSO legal determination. There are considerations whether reliability studies are conducted in research settings with trained raters operating under strict procedures and “field reliability” focused on real world clinical practitioners where reliability is comparatively suboptimal (Aboraya, 2007).

In research settings, for example, the interrater reliability of ICD-10 schizophrenia diagnoses using a diagnostic checklist for 100 subjects yielded *k* = 0.60; when diagnoses were amalgamated into a diagnostic entity of schizophrenia-spectrum disorders, *k* = 0.98 (Jakobsen et al., 2005). Interrater reliability for psychiatrists using the Composite International Diagnostic Interview for diagnoses of schizophrenia yielded *k*-values of 0.59 and 0.56 for DSM-IV and ICD-10, respectively (Cheniaux et al., 2009). An interrater reliability study of the schizoaffective disorder diagnosis using the Composite International Diagnostic Interview with 150 patients yielded a Cohen's *k* = 0.22 (Maj et al., 2000). Interrater reliability for DSM-5 field trials yielded poor level of agreement: schizoaffective disorder (*k* = 0.50), schizophrenia (*k* = 0.46), and attenuated psychotic symptoms syndrome (*k* = 0.46; Freedman et al., 2013). The high levels of error in these findings suggest serious problems with application of diagnostic criteria by judges.

Interrater reliability of clinical symptoms is somewhat more encouraging. Interrater reliability using the Swedish version of the Structured Clinical Interview for the Positive and Negative Syndrome Scale (SCI-PANSS) yielded intraclass correlations of 0.98–0.99 for the Positive Symptom Scale, and 0.83–0.90 with the Negative Symptom Scale. The General Psychopathology Scale yielded intraclass correlation coefficients of 0.95–0.98 (Lindström et al., 1994). Interrater reliability of a shortened 6-item PANSS in a sample of schizophrenic in- or out-patients, yielded ICCs in the good range (ICC = 0.74). ICCs for the six individual scale items ranged from 0.45 to 0.76 (Kølbæk et al., 2018). Studies of the PANSS utilizing taped observations ranged from 0.56 to 0.99 for the Positive Symptom Scale, and 0.20 to 0.90 for the Negative Symptom Scale. Total PANSS scores ranged from 0.66 to 0.71 for taped interview observations (Crittenden et al., 2009). A literature survey of interrater reliability for diagnosis of delusions in general found substantial agreement using a variety of structured interviews and the PANSS, ranging from 0.64 to 0.93. The diagnosis of bizarre delusions, however, was rather poor falling into the 0.41–0.60 range (Bell et al., 2006).

These findings indicate that the use of structured clinical measures in research settings, yields marginally reliable clinical diagnoses. The field reproducibility of diagnostic impressions is therefore weak and in some studies less than chance. Evaluation of clinical symptoms fare better than diagnostic judgments. These studies suggest that degree of confidence in clinical diagnosis under field conditions is much lower. Tempering any overly favorable assessment of the reliability of psychiatric diagnosis, however, Vanheule et al. (2014) note that since DSM-III, norms for evaluating ICC and *k* coefficients have relaxed considerably. They note that DSM-5 field trials used “unacceptably generous” norms, and conclude that diagnostic reliabilities in 2013 are not notably better than 1974.

Beyond limited field trials, scientific assessment of DSM psychiatric disorders have not been undertaken. The majority of DSM-5 diagnostic categories were not tested at all: the DSM-5 counts 347 disorder categories, but kappa coefficients were calculated for only 20 conditions (6%). Of those categories only 14% had a good or very good reliability, which means that only 4% of the DSM-5 categories have been shown to have acceptable reliability. Since the inter-rater reliability of the majority of the DSM-5 categories remains untested, this raises serious questions about diagnostic reliability in the clinical assessment of MSO evaluations for diagnoses and to a lesser extent for clinical features.

### Criminal Responsibility Opinion-Making

Hawaii's three panel system for court-ordered forensic examinations has been intensively studied over the past 10 years since it offers a unique laboratory to study inter-rater reliability and examiner and judicial consensus. In felony cases, Hawaii uses the Model Penal Code (MPC) CR language focused on cognitive and volitional capacity. A Hawaii study of 150 independent CR reports conducted by court-appointed three examiner panels yielded “fair” levels of agreement (ICC = 0.51; Fuger et al., 2013). In 23 cases (69 reports, 46%), all three examiners achieved consensus. In 26 cases (78 reports, 52%), at least two evaluators reached consensus: Psychiatrists and community-based psychologists (CBP) reached a “fair” level of agreement (ICC = 0.57, *p* < 0.01). Community-based psychologists and court-based examiners reached a “fair” level of agreement (ICC = 0.54, *p* < 0.01). Psychiatrists and court-based examiners reached consensus with “fair” levels of agreement (ICC = 0.42, *p* < 0.01). A study of Hawaii's court-appointed three panels using a separate sample aggregated agreement coefficients for CR yielded an ICC of 0.51. Average pairwise Cohen's *k* was 0.391 (Guarnera et al., 2017).

In a second more rigorous study examining five types of reliability coefficients in 150 cases in a non-crossed data measurement design, reliability of CR decisions in panels of three independent court-appointed examiners was marginal (*k* = 0.39; Acklin and Fuger, 2016). A field reliability study examining CR decision making in three examiner panels including CBP, community-based psychiatrists (PSY), and court-based psychologists (DOH) found Fleiss's *k* = 0.39. Average pairwise Cohen's kappa was *k* = 0.39. Average pairwise Cohen's *k* between PSY and CBP was 0.32, PSY and DOH was 0.45, and CBP and DOH was 0.40. Criminal responsibility field reliability studies in other jurisdictions have found similar results. Meta-analytic procedures and study space methodology applied to field reliability of insanity opinions found level of agreement for sanity opinions (*k* = 0.41; Guarnera et al., 2017). These reliability coefficients fall into “poor-fair” range of agreement and reflect lower levels of agreement than competency to stand trial decisions (*k* = 0.49).

It should not be surprising that levels of agreement in CR judgments are poor given the complexity of the evaluations, retrospective nature of MSOs, discretionary variability, and availability of information utilized, previously discussed unreliability in diagnostic classification, variability in evaluator training and skill, and of primary importance, the widespread reliance of unstructured clinical judgment (Monahan, 2008; Faust and Ahern, 2012; Sutherland et al., 2012; Hart et al., 2016).

Summarizing, CR evaluations require a clinical MSO evaluation and formulation of a forensic judgment based on the collected and aggregated data. Diagnostic studies range from poor to good for some psychosis-related constructs such as positive symptoms, including delusions. Level of agreement for forensic judgments indicates poor reproducibility and high level of error in CR decision-making (Acklin et al., 2015). These errors are not inconsequential. In considering these elements of CR evaluations—clinical status at the time of the offense, including psychiatric diagnosis, and forensic judgments—these findings highlight concerns about methodology, standardization, decision models, and presence of biases and error (Neal and Grisso, 2014a,b).

This survey of the CR behavioral science decision making identifies concerns about the reliability and objectivity of opinions proffered to courts of law. These shortcomings demand methodological reform in practice standards and methodological rigor in the performance of forensic mental health evaluations (National Research Council, 2009). In the sections that follow, in response to calls from critics of unstructured clinical judgment, an alternative decision method is described using SPJ (Hart et al., 2017) for data collection and a mechanical decision model for data aggregation will be described.

## Structured Professional Judgment Methods in Forensic Psychology

The emergence of SPJ (Monahan, 2008; Hart et al., 2016) as a corrective for unstructured clinical judgment (and an alternative to rigid non-discretionary actuarial algorithmic decision models) has been applied to various risk assessment methodologies (notably violence and sex offending; e.g., Sutherland et al., 2012). Structured professional judgment utilizes a model based on empirical “guidelines” that form a conceptual and empirical structure for risk assessment and management. It is proposed here that the SPJ model may make a significant contribution to standardizing, organizing, and disciplining the assessment and decision making process in non-risk assessment forensic psychology.

Structured Professional Judgment has become synonymous with a methodology developed by Hart et al. in forensic risk assessment (Hart et al., 2016). Hart et al. (2016) describes several steps in the SPJ procedure:

1. identifying the presence of a priori risk factors (“guidelines”),
2. gathering information,
3. considering the relevance of risk factors that are present,
4. developing a formulation of risk based on findings,
5. developing a risk management plan, and
6. communicating summary judgments.

Hart address the interpretive “gap” between steps 3 and 4 by specification or guidance for formulation of risk and scenario planning (Hart et al., 2016, p. 653). This aspect of SPJ methodology has been criticized for relying on informal data aggregation and mixing algorithmic and clinical judgment (Hilton et al., 2006; Falzer, 2013).

Empirical studies of risk assessment methodologies have been controversial. A meta-analysis conducted by Hanson and Morton-Bourgon (2009) obtained a rank order for decision methods they analyzed. Actuarial procedures were the most accurate overall, followed by the hybrid method mixing clinical and actuarial methods, SPJ, and finally unstructured clinical judgment. Guy (2008) examined comparative performance of risk assessment methodologies, unstructured clinical, actuarial, and SPJ. Guy's (2008) evaluation, cited in Guy et al. (2015) of all available research on the predictive validity of SPJ instruments found, consistent with previous research, that “unstructured approaches were significantly less strongly related to violence that were structured approaches either actuarial or SPJ” (p. 53). Guy et al. (2015) conclude that “empirical findings provide strong support for the SPJ model, that SPJ is at least as or more accurate than actuarial instruments, and unstructured clinical prediction” (p. 53). Findings from other meta-analyses comparing effects from SPJ and actuarial measures and violence risk assessment generally found that effect sizes for scores from actuarial tools were similar to those derived from SPJ measures (Chevalier, 2017). While there is no clear evidence that one approach is superior to the others, application of an evidence-based structure appears to improve accuracy relative to unstructured decision-making.

In conducting a CR case, after a thorough assessment of background, offense information, and clinical examination of the defendant, clinical and forensic assessment instruments are available to assist in the process of collecting, structuring, and organizing assessment evidence. Clinical description and diagnosis of the defendant's MSO, for example, may be usefully assessed using reliable instruments such as the Positive and Negative Symptoms Scale (PANSS; Kay et al., 1987). The PANSS has an associated structured clinical interview (SCI-PANSS; Opler et al., 1992), useful in developing predictors related to delusions, hallucinations, hostility, and potential for violence. The Rorschach Test has shown powerful capacity to identify psychotic thinking (Acklin, 1992, 1999, 2008; Kleiger and Khadivi, 2015; Kleiger, 2017). In the data-opinion linkage, the Rogers Criminal Responsibility Assessment Scales (R-CRAS, 1984; Rogers and Shuman, 2000) are a very useful data aggregation measure with a built in CR decision model. The R-CRAS is specifically designed for CR assessments that assist in systematically assessing relevant factors applying behaviorally-anchored rating scales for linking evaluation findings to CR legal standards. The focus of this paper is the application of SPJ violence risk assessment model to forensic assessment of CR, by substituting empirically-based *a priori* “postulates” for risk guidelines, utilizing structured clinical and forensic assessment instruments, formal aggregation of evidence, and *post-hoc* hypothesis testing.

## An SPJ Decision Model for Criminal Responsibility

**Figure 1:**
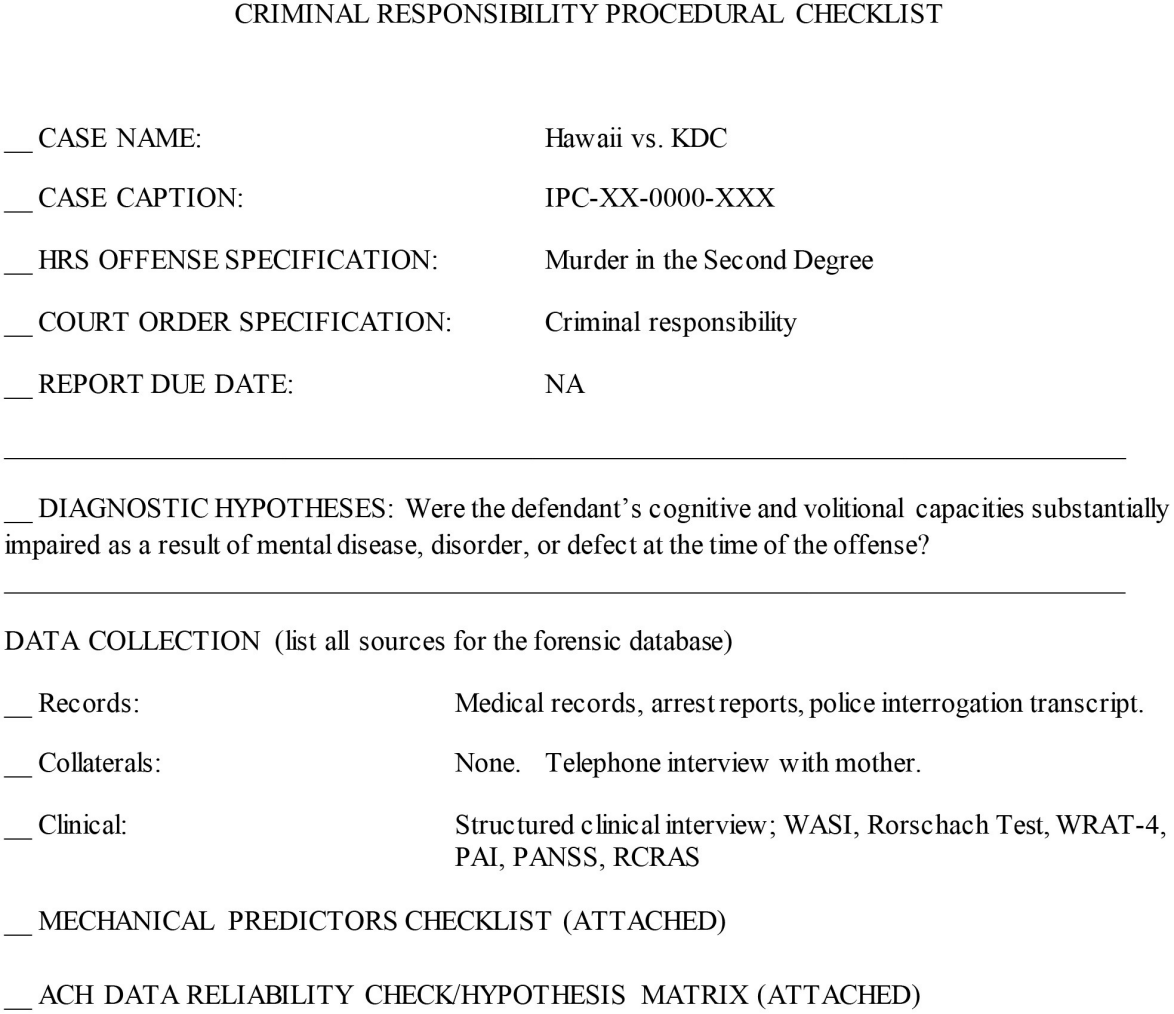
Criminal responsibility procedural checklist.

**Figure 2:**
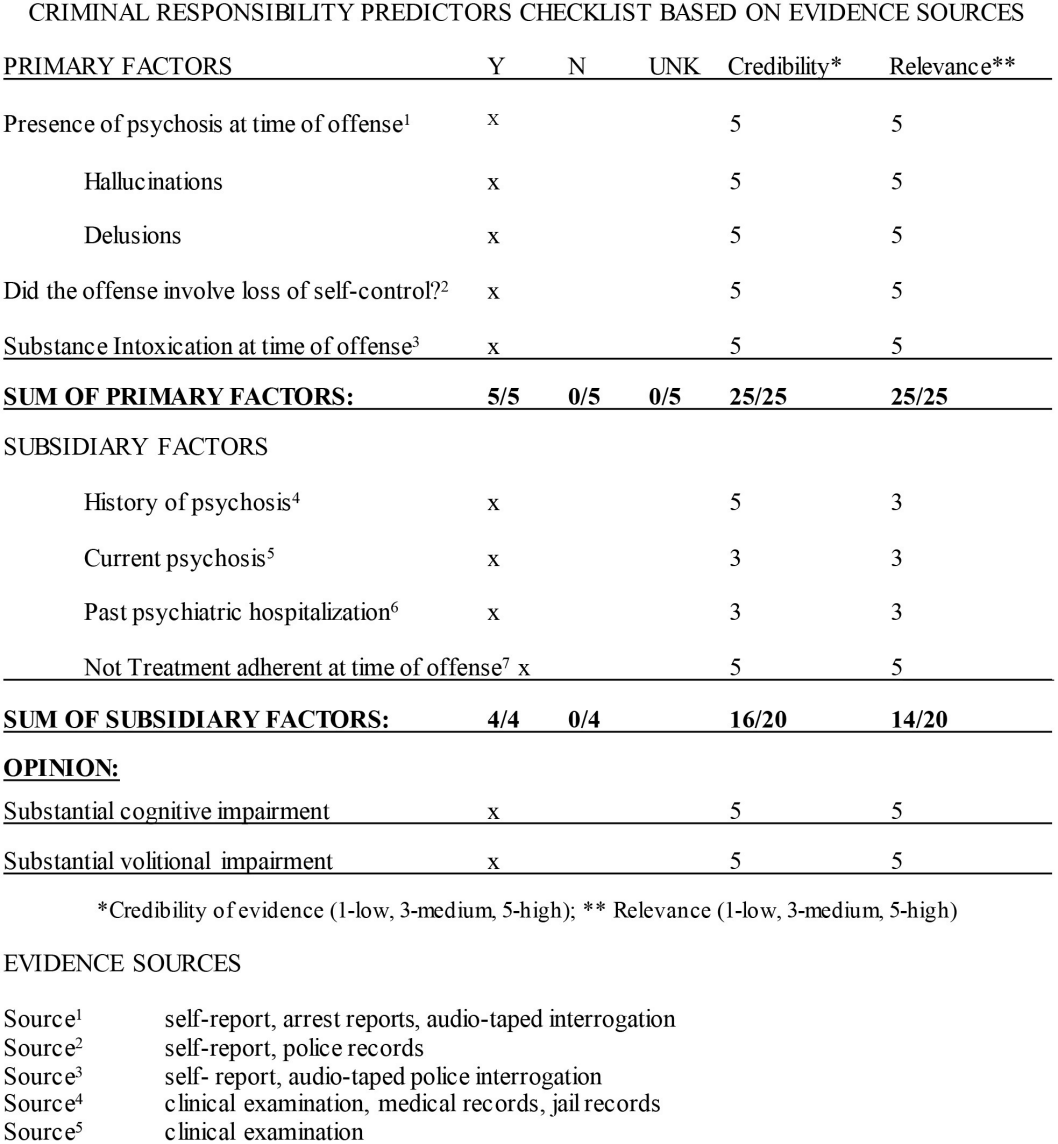
Criminal responsibility predictors checklist based on evidence sources. *Credibility of evidence (1—low, 3—medium, 5—high); **Relevance (1—low, 3—medium, 5—high). Evidence sources: ^1^self-report, arrest reports, audio-taped interrogation; ^2^self-report, police records; ^3^self-report, audio-taped police interrogation; ^4^clinical examination, medical records, jail records; ^5^clinical examination; ^6^medical records; ^7^self-report, collateral report. Opinion: The decision model indicates that the criminal conduct involved a loss of behavioral self-control due to acute Methamphetamine Intoxication and Methamphetamine-induced psychotic disorder. There are strong suspicions that defendant was psychotic *prior* to the offense due to his previous chronic ice use, clinical history, and report of collaterals. It is more likely than not that the defendant's cognitive and volitional capacities were substantially impaired at the time of the offense as a result of methamphetamine-induced psychotic disorder and acute methamphetamine intoxication.

**Figure 3:**
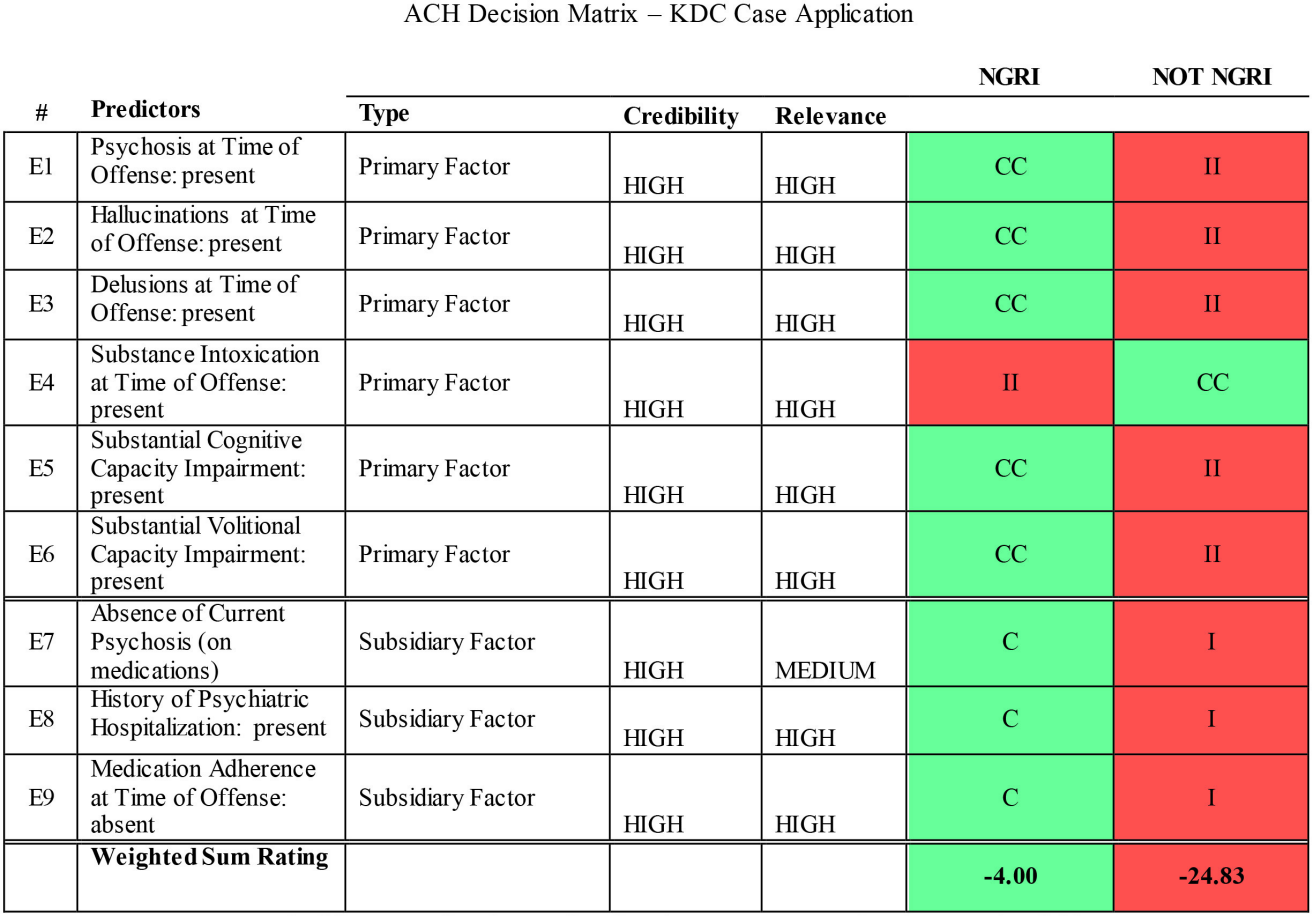
ACH decision matrix—KDC case application. CC, very consistent; C, consistent; N, neutral; NA, not available; II, Very Inconsistent; I, inconsistent. The primary hypothesis based on the court's question: Were the defendant's cognitive and/or volitional capacities substantially impaired due to mental disorder at the time of the offense (NGRI)? The logic of ACH is to *disprove* the primary hypothesis (i.e., prove the alternative hypothesis). The alternate hypothesis is that defendant's cognitive and/or volitional capacities were *not* impaired at the time of the offense (not NGRI). The weight of the evidence is against not NGRI (weighted sum rating = −24.83). Conclusion: The best explanation based on the available evidence is that KDC was not criminally responsible (Not NGRI) at the time he committed the offense.

**Figure 4:**
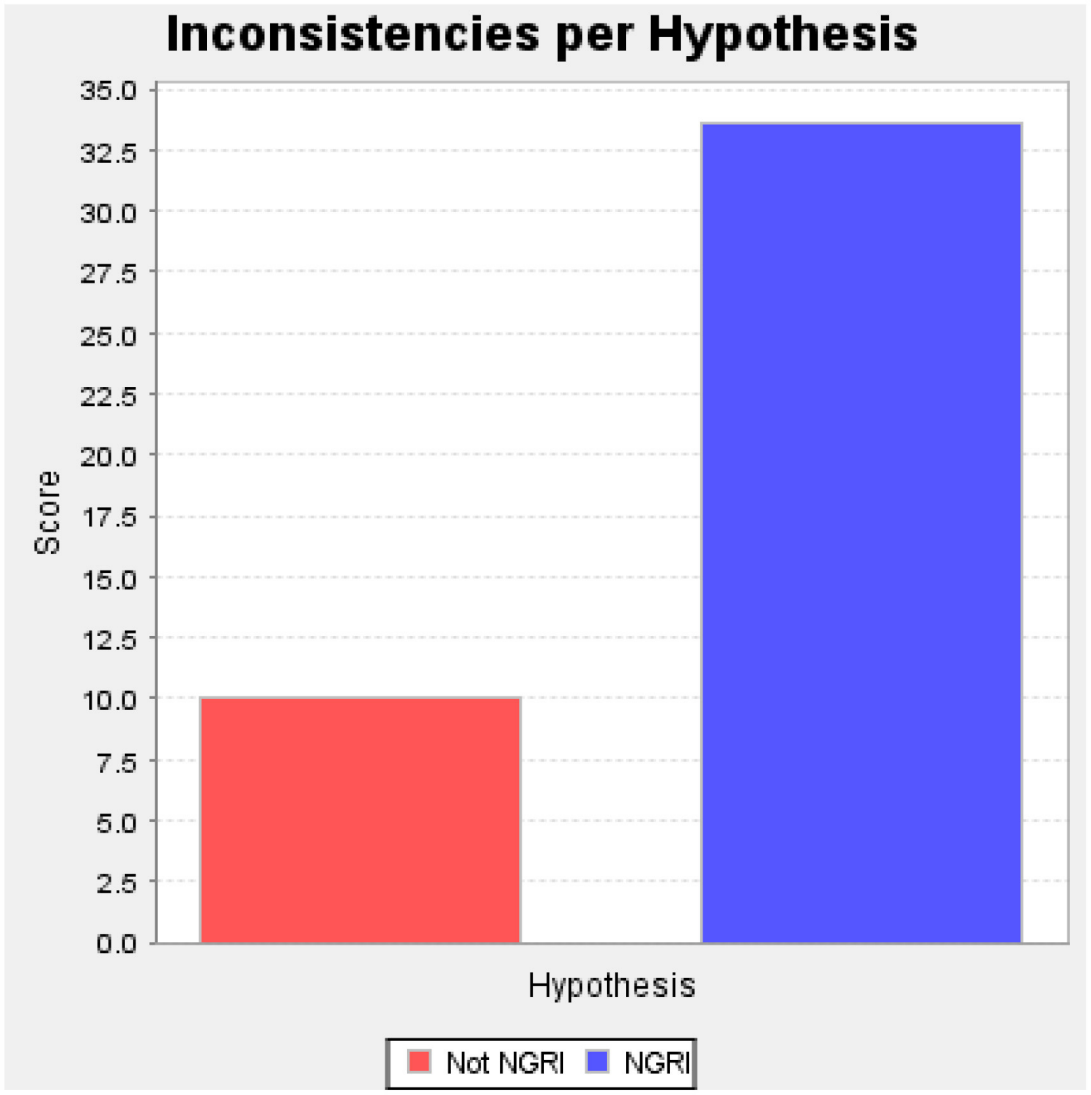
Graphic representation of KDC case ACH.

Scientific method involves two primary functions: disciplined data collection and interpretation (Sawyer, 1966; Faust and Ahern, 2012). Structured data collection—including fixed or prespecified vs. variable procedures—and structured prediction make independent contributions to accuracy (Sawyer, 1966; Faust and Ahern, 2012, p. 158). Checklists have been advocated for standardizing report procedures and format (Witt, 2010). The model described here proposes the use of procedural checklists to structure and standardize data collection (Figure 1), structured aggregation and weighing of a priori empirical predictors according to SPJ principles, including the relevance and credibility of collected (Figure 2), and *post-hoc* analysis of final opinions (Figures 3, 4).

Research across a range of fields has demonstrated improved decision making accuracy for structured over holistic methods (Monahan, 2008; Faust and Ahern, 2012; Kuncel et al., 2013; Neal and Grisso, 2014a; Hart et al., 2016). Unstructured clinical judgment is degraded because judges are inconsistent in how they weigh cues, and combine, and weigh information across targets (Kuncel et al., 2013). Variable attention to salient cues and inconsistent application of weights yields inferior predictive power compared to structured combination of fixed predictors. The innovation proposed here is an application of a SPJ decision model to MSO data and forensic opinions in an effective, user-friendly procedure. In contrast to fears that a structured prediction model is rigid, cumbersome, or overly technical (which have been identified as sources of clinician resistance to structured methods; Vrieze and Grove, 2009; Lilienfeld et al., 2013), the proposed model focuses on the reduction of data collection to the most powerful predictors central to the legal standard using a simple worksheet format (Meijer et al., 2020; see Figure 2).

For the predictor aggregation task, simple actuarial formulae based on a few variables equal or exceed the level of accuracy achieved through clinical judgment (Dawes et al., 1989). Further, identification of robust predictors “is more important than trying to determine differential weights or discern patterns among those variables” (Faust and Ahern, 2012, p. 201). Simply adding variables together using linear composites or assigning equal unit weights is superior to attempting to optimize weights (Faust and Ahern, 2012).

Additionally, the model described here applies a *post-hoc* analysis to systematically control for confirmatory bias, using an evidence weighing model: the Analysis for Competing Hypotheses (ACH; Heuer, 1999, 2005). The ACH is a structured analytic technique developed by the CIA as a tool for analyzing complex, ambiguous data under conditions of uncertainty in intelligence analysis. The ACH matrix contains the set of evidence-based predictors derived from the scientific literature, and case specific clinical postulates which are assigned weights in a decision matrix. A computer interface allows for manipulation of variables and their respective weights. The application of the ACH to a structured clinical judgment decision model corrects concerns about unstructured clinical data aggregation and judgment. Since the methodology is designed to specifically counter confirmation biases, the model analyzes data which disconfirms the primary hypothesis (namely, that the defendant is not criminally responsible). It is also useful for an assessment of the reliability and credibility of evidence. The matrix reduces the gap between clinical and forensic data and opinions by systematically testing hypotheses in the final stage of the CR opinion process (Figure 3).

Based on the proposition that both structured data collection and mechanical prediction make independent contributions to accuracy (Faust and Ahern, 2012, p. 158), the proposed CR structured prediction model advocates the use of:

a) checklists for structured application for procedures (Figure 1);
b) structured data gathering organized around a priori predictors^2^;
c) data aggregation utilizing predictors using an unweighted aggregation model (Figure 2); and
d) structured analytic techniques to consider evidence sources, credibility and relevance and *post-hoc* hypothesis testing using the ACH (Heuer, 1999, 2005, Figure 3).

The application of the SPJ model to CR forensic decision-making provides a systematic, individualized, and evidence-based exposition and analysis of factors supporting the forensic judgment.

## Case Study

KDC was referred by his public defender for a CR^3^ and competency to stand trial evaluation. KDC is a 21-year old man accused of murdering 46-year old IL, a visitor to his house, by stabbing him multiple times in the chest. He killed IL as they were sitting on the lanai outside KDC's home (his mother was in an adjoining room watching TV), stabbing him suddenly and violently without warning. KDC and IL had smoked crystal methamphetamine (“ice”) immediately before the stabbing, and also the day before. KDC immediately fled the scene in his mother's car “to go into the mountains” but turned himself into police several hours later after his cousin told him to “man up.” IL was living in a homeless camp behind the house and he was a frequent visitor to the residence. IL knew KDC's mother and she sometimes gave him food. IL was deaf. According to KDC, IL would come over when he was not home, or when he was sleeping, and “invade” KDC's space. He showed “disrespect” by walking in the house with mud on his slippers. KDC thought IL was stealing from him. KDC admitted there had been a couple of previous hostile encounters with IL but no physical violence. He admitted that he was frightened by IL, who carried homemade weapons made of bicycle parts.

When asked why he had killed the decedent, he could not describe his thoughts or feelings, only that “something had built up” inside of him and then “I just did it.” Immediately before the stabbing, he had overheard IL telling his mother that someone was hanging dogs and cats in the woods behind the house, which angered him because it upset his mother. Around 17:00 h the decedent walked to the back lanai by himself to smoke “clear.” KDC discussed what IL had said to his mother. His mother thought KDC was acting “strange.” From the police interrogation transcript, when asked why he killed him, KDC reported that “I just got weak.” He denied feeling angry at the decedent or frustrated, “…just weak, and I could not feel my arms.” The detective wrote, “He said it was a buildup of the decedent's behaviors and he just got weak and could not tolerate them anymore” and he stabbed IL multiple times in the chest.

The Emergency Department medical clearance conducted at a local hospital several hours after the stabbing indicate altered mental status (i.e., psychosis). The police interrogation and closing investigative reports conducted the next day demonstrated bizarre thinking. Jail mental health records described psychotic mentation and he was started on antipsychotic medications. By the time of the examination his symptoms had remitted. Both police and ER medical reports described his behavior as “strange,” that his affect was odd, and that he was talking non-sensically. KDC made an audio-recorded and transcribed statement to a police detective the next day describing the stabbing in detail. He said he was afraid that IL was going to kill him and his mother, if he did not kill him first. The mother stated that she was afraid of KDC in the days prior to the stabbing, that his thinking and behavior were strange and frightening, thinking that he might “erupt” at any minute. She reported that he had been up at night, that his manner was agitated and suspicious, that he was hearing “things,” and looking outside the house saying that “someone was out there.” She had observed KDC's psychotic behavior many times over a period of months. She was extremely worried that he had stopped going to treatment, was not taking his antipsychotic medications and that something bad could happen.

KDC has a well-documented history of multiple psychiatric hospitalizations, the great majority of which involved flagrantly bizarre behavior (walking in traffic, raving in public) associated with positive methamphetamine toxicology screens. The record includes records from several psychiatric hospitalizations for methamphetamine-induced psychotic disorder^4^. Prior to the time of the stabbing, he was intermittently working construction labor and participating in an IOP while on probation for earlier drug possession (marijuana) and weapons charges (carrying a knife). His mother said few weeks before he stopped going to work, attending the program, and taking his medications (Seroquel 400 mg at bedtime). His behavior deteriorated. On clinical exam, he had been in jail about 4 months. He had been prescribed Seroquel in jail but stopped taking it the previous week. He had been seen by psychiatry and mental health who described no current clinical psychosis. He was housed in the general population.

A psychological assessment indicated borderline intellectual abilities, no current clinical signs of psychosis, including negative findings on a Rorschach Test. His PAI was invalidated due to over reporting. The case material and clinical assessment material were coded using the PANSS (at time of offense and at assessment) and RCRAS. The PANSS profile indicated the presence of positive symptoms: hallucinations and or delusions at the time of the offense. The RCRAS indicated the offense involved loss of control under the influence of acute methamphetamine intoxication and chronic methamphetamine psychosis (see note 4 below). At the time of the forensic examination, his symptoms had remitted but to cessation of methamphetamine and medication compliance.

## Results

Figures 1–3 illustrate the procedural checklist, decision model worksheet, and hypothesis testing matrix. Figure 1 is the procedural checklist. Figure 2 illustrates the structured predictive model. The aggregated decision rule makes the elements of the decision explicit. Figure 3 illustrates the ACH *post-hoc* hypothesis testing matrix. The matrix has summary weighted scores based on degree of consistencies and inconsistencies with the primary and alternate hypotheses. Individual a priori predictors and evidence sources are rated with respect to predictor credibility and relevance. The logic of ACH is examination of data which *disprove* the primary hypothesis (that KDC was CR). Figure 4 illustrates a graphic representation of the weight of the evidence.

In terms of the Hawaii two-prong insanity statute, the SPJ aggregation model and review of evidence presented in Figure 2 indicates that the defendant's cognitive abilities and volitional capacities were substantially impaired by acute methamphetamine intoxication and pre-existing methamphetamine-induced psychosis. Under Hawaii statutes, voluntary intoxication does not excuse criminal conduct and is not eligible for an insanity defense.^5^ However, a recent Hawaii Supreme Court decision reiterated the doctrine of permanent or “settled insanity” (the presence of persistent psychosis after acute drug intoxication has stopped) and examiners and judges had to consider whether the defendant was psychotic *prior* to the acute intoxication and commission of the crime. Given the weight of the predictors and the evidence, *post-hoc* hypothesis testing indicates a high degree of confidence may be placed in the findings, and the opinion may be proffered to a reasonable degree of psychological probability.

Based on the credibility and relevance ratings, the evidence database is sufficient to render legally admissible opinions under the Hawaii Rules of Evidence (which follow the Federal Rules of Evidence on the admissibility of expert testimony). The evidence aggregation model (Figure 2) yielded five positive and zero negative results on the primary factors based and four positive and zero negative results on the subsidiary factors on credible and relevant information, yielding the opinion that KDC's cognitive and volitional capacities were substantially impaired by acute methamphetamine intoxication and pre-existing methamphetamine-induced psychosis.

The final CR decision derived from the SPJ approach parallels summary risk ratings (SRR) in violence risk assessment instruments, although it does not yield a numerical value. The final CR decision may be applied to any clinically derived final judgment to yield a total score (based on the frequency weights from the primary and subsidiary predictors; Figure 2). While most SPJ risk measures yield a SRR of low, moderate, or high, in the model advocated here, based on abductive reasoning (Ward and Haig, 1997), the evaluator reaches a decision with a low, moderate, or high degree of confidence in the decision based on the explanation that best supports the opinion.

The SPJ decision model has insufficient discrimination to address the challenging question whether KDC may have been suffering from a primary psychotic disorder (e.g., schizophrenia) vs. persistent methamphetamine-induced psychosis at the time of the offense (McKetin et al., 2017; Wearne and Cornish, 2018)^6^. The record does indicate the presence of a pre-exiting psychosis. The decision model explicitly describes the evidence basis, method of analysis, decision making rule, with a hypothesis testing procedure (ACH, Figures 3, 4) to control for confirmation bias. These procedures integrate a local clinical scientist orientation to forensic decision-making (Stricker and Trierweiler, 1995) utilizing SPJ principles.

## Conclusion

This paper is a pilot description of a structured prediction model for CR opinions applying the principles of SPJ and a transparent data aggregation procedure. Key issues in the CR prediction model include the (1) use of checklists to outline and standardize procedures; (2) structured data collection focused on robust *a priori* predictors, (3) reliable retrospective clinical diagnosis at the time of the offense, (4) structured data aggregation, (5) linkage between clinical, functional, and legal elements, and (6) *post-hoc* hypothesis testing of case data. The value of the structured decision model is the *a priori* specification of robust predictors, weighting of evidence using relevance and credibility ratings, application of predictors to case material, and a *post-hoc* hypothesis testing procedure to verify opinions, and reduce confirmatory bias. The model externalizes what forensic clinicians already do in an explicit structured procedure. The methodology, based on robust SPJ principles, adheres to a core empirical foundation. A similar methodology may be applied to other forensic decision-making tasks, e.g., competency to stand trial or post-acquittal conditional release.

Judgment is inevitable in the forensic decision making process. The application of SPJ methodology, including use of empirical guidelines, and rigorous hypothesis testing closes but does not eliminate the gap between evidence and decision. The SPJ methodology organizes and structures the assessment process. The aggregation of empirical predictor model yields sturdy evidence-based decisions. The ACH provides *post-hoc* control for hypotheses testing and control for confirmation biases. It makes explicit the strengths and weaknesses of the evidence and decision model, assists in the use of the model as probative evidence, and reduces but does not eliminate the gap between data and inference making.

## Summary and Future Research Directions

This report provides a conceptual foundation for SPJ decision making for forensic CR opinions. The aim of the project is the “translation and dissemination of the science of risk assessment into the field where such evaluations regularly occur” (Guy et al., 2015, p. 75). The paper describes a pilot project applying the SPJ model to an actual forensic case. Further steps in developing the empirical properties of the model are necessary, including testing with practitioners, reliability and accuracy assessments, and ease with which the model can be acquired by field practitioners. This preliminary model will require empirical testing to demonstrate that forensic practitioners can be trained and apply the model to a set of insanity cases utilizing identical case information, comparing conventional unstructured clinical and SPJ methods. This will be necessary to test whether the application of a SPJ decision model accuracy of judgements and enhances level of inter-rater agreement and provides more accurate CR opinions. The model can be applied to other types of forensic mental health evaluations, (e.g., competence to stand trial, post-acquittal conditional release).

## Data Availability Statement

The raw data supporting the conclusions of this article will be made available by the authors, without undue reservation.

## Author Contributions

All authors listed have made a substantial, direct and intellectual contribution to the work, and approved it for publication.

## Conflict of Interest

The authors declare that the research was conducted in the absence of any commercial or financial relationships that could be construed as a potential conflict of interest.

## Footnotes

^1^Whether forensic psychologists should proffer ultimate opinion testimony is controversial (Rogers and Ewing, 2003; Buchanan, 2006; Melton et al., 2007). Following John Hinckley's attempted assassination of Ronald Reagan and subsequent acquittal, Federal Rules of Evidence Rule 704 was revised in 1984 to prevent expert testimony on mental state at the time of the offense. In Hawaii, the criminal courts order experts to provide ultimate opinions in CR examinations.

^2^The predictors are derived from the forensic behavioral science research literature (Warren et al., 2004) focused on the strongest empirical indicators for criminal responsibility tied to Hawaii's two-pronged insanity statute (substantial impairment in cognitive and/or volitional capacities at the time of the offense; Hart et al., 2016).

^3^Hawaii's two prong insanity statute is based on the ALI Model Penal Code. Hawaii Revised Statutes §704–400: Physical or mental disease, disorder, or defect excluding penal responsibility. A person is not responsible, under this Code, for conduct if at the time of the conduct as a result of physical or mental disease, disorder, or defect the person lacks substantial capacity either to appreciate the wrongfulness of the person's conduct (cognitive capacity) or to conform the person's conduct to the requirements of law (volitional capacity).

^4^A substantial minority of chronic methamphetamine users develop a psychosis resembling paranoid schizophrenia (described as a toxic paranoid-hallucinatory state) which persists after cessation of acute intoxication (Sato, 1992; Acklin, 2016).

^5^HRS 702-230: Evidence of self-induced intoxication of the defendant is not admissible to negative the state of mind sufficient to establish an element of the offense. Self-induced intoxication means intoxication caused by substances which the defendant knowingly introduces into the defendant's body, the tendency of which to cause intoxication the defendant knows or ought to know. However, a recent Hawaii Supreme Court decision (Hawaii vs. Abion 146 Hawaii 230) reiterated the doctrine of permanent or “settled insanity” (the continuing presence of psychosis after drug use has stopped) and that examiners and judges had to consider whether the defendant was psychotic *prior* to using drugs. Voluntary intoxication is not a defense unless it produces permanent insanity.

^6^McKetin et al. (2018) examined 554 current methamphetamine users using the Composite International Diagnostic Interview finding a distinction between schizophrenia and methamphetamine-related psychotic symptoms both in terms of propensity to experience psychotic symptoms and symptom profile (p. 1).
